# Gemcitabine plus cisplatin versus gemcitabine alone in the treatment of pancreatic cancer: a meta-analysis

**DOI:** 10.1186/s12957-016-0813-9

**Published:** 2016-02-29

**Authors:** Guoqing Ouyang, Zhipeng Liu, Shengfu Huang, Qianglong Li, Li Xiong, Xiongying Miao, Yu Wen

**Affiliations:** Department of General Surgery, The Second Xiangya Hospital,Central South University, No.139 Renmin Road, Changsha, 410011 Hunan People’s Republic of China

**Keywords:** Pancreatic cancer, Gemcitabine, Cisplatin, Meta-analysis

## Abstract

**Background:**

Pancreatic cancer ranks as the fourth leading cause of cancer-related mortality in the USA. And gemcitabine has been the standard of care for advanced pancreatic cancer. However, a combined use of gemcitabine plus cisplatin (GemCis) has shown promising efficacies in pancreatic cancer patients. Here, system review and meta-analysis were performed to compare the efficacy and safety of GemCis versus gemcitabine (Gem) alone in the treatment of pancreatic cancer.

**Methods:**

The databases of MEDLINE (PubMed), EMBASE, and Cochrane Library were systematically searched for retrieving the relevant publications prior to 31 September 2014. The primary end point was overall survival (OS) and secondary end points included 6-month survival, 1 year survival, overall response rate (ORR), clinical benefit rate (CBR), time to progression/progression-free survival (TTP/PFS), and toxicities.

**Results:**

A total of nine randomized controlled trials involving 1354 patients were included for systematic evaluations. Overall, as compared with Gem alone, GemCis significantly improved the 6-month survival rate (relative risk (RR) = 1.303, 95 % confidence interval (CI) 1.090–1.558, *P* = 0.004), ORR (RR = 1.482, 95 % CI 1.148–1.913, *P* = 0.003), PFS/TTP (hazard ratio (HR) = 0.87; 95 % CI 0.78–0.93, *P* = 0.022), and the overall toxicities (RR = 2.164, 95 % CI 1.837–2.549, *P* = 0.000). However, no significance difference existed in overall survival (HR = 0.90, 95 % CI 0.80–1.42, *P* = 1.02), 1-year survival rate (RR = 0.956, 95 % CI 0.770–1.187, *P* = 0.684), and CBR (RR = 0.854, 95 % CI 0.681–1.072, *P* = 0.175). As for grade III/IV toxicity, seven kinds of toxicities were higher in the GemCis group. However, no significant inter-group statistical differences existed in the incidence of leukopenia, thrombocytopenia, or diarrhea.

**Conclusions:**

Despite a higher incidence of three-fourths toxicity, GemCis offers better outcomes of ORR, PFS/TTP, and 6-month survival, which indicates GemCis may be a promising therapy for pancreatic cancer.

## Background

As the fourth leading cause of cancer-related death in the USA [1], pancreatic cancer is one of the most lethal malignancies due to its diagnostic difficulties. Most patients have been in an advanced stage or a metastatic disease when diagnosed [2]. And 50 % of them were metastatic, 30 % locally advanced, and only 20 % resectable tumors [3]. Most cases had already progressed beyond the point of surgical resectability. As reported by the American Cancer Society, the median survival for locally advanced pancreatic cancer is only 9–15 months; however, in metastatic patients, this may fall to 3–6 months and its overall 5-year survival is no more than 4 % [4].

The management modalities of pancreatic cancer may be summarized as surgical resection, chemotherapy, radiotherapy, chemoradiation, etc. Despite an availability of different managements, the outcomes of pancreatic cancer patients were similar with a median survival of 20 to 22 months [5]. So improving disease symptoms and quality of life and offering better clinical benefits have become the key end points of palliative chemotherapies [6].

In 1997, gemcitabine (Gem) became a de facto standard of care for advanced pancreatic cancer. As compared to 5-fluorouracil, it modestly increased overall survival and offers better clinical benefits [7]. Gemcitabine has consistently resulted in a median overall survival of 5–7 months and approximately 20 % increase in 1-year survival rate in metastatic patients [8]. Though with a modest clinical benefit, it failed to improve much of the dismal prognosis. For achieving better clinical efficacies, multiple gemcitabine-based schemes have been attempted in clinical setting.

As a valid inducer of apoptosis in pancreatic cancer cells, cisplatin is among the most effective and widely used chemotherapeutic agents [9]. A combination of gemcitabine plus cisplatin (GemCis) was synergistic so that it worsened DNA damage [10, 11].

A previous meta-analysis presented by Xie et al. [12] in 2006 suggested that the gemcitabine monotherapy has remained as a standard treatment for pancreatic cancer, while the combination regimen is not recommended. Their meta-analysis has included some meeting abstracts whose full texts could not be acquired, so that their conclusion is somewhat weak. And the search was restricted up to March 2005. For updating and strengthening, the authors performed another meta-analysis of recently published trials comparing GemCis with Gem alone with regards to median OS, 6-month and 1-year survivals, overall response rate (ORR), and clinical benefit rate (CBR).

## Methods

### Searching strategy

From inception to 31 September 2014, comprehensive electronic searches were performed within the database of EMBASE, MEDLINE (PubMed), Cochrane Central Register of Controlled Trials, China National Knowledge Infrastructure (CNKI). The relevant medical subject heading and free words included “gemcitabine,” “gemzar,” “cisplatin,” and “pancreatic cancer/carcinoma/adenocarcinoma.” And the searching languages were limited to English and Chinese.

### Date extraction and quality assessment

Two authors (i.e., GQOY and ZPL) independently abstracted the data from each study. Any discrepancies between the two reviewers were resolved by consensus and discussion. The following parameters were extracted: (1) publication and first author’s name; (2) patients, characteristics, number of eligible patients, treatment arm, study design, and follow-ups; (3) treatment outcome, such as OS, 6-month and 1-year survival rates, ORR, CBR, TTP/PFS, and toxicities.

### Criteria for inclusion and exclusion

The studies included in this meta-analysis should fulfill the following inclusion criteria: (1) cytologically or histologically confirmed advanced stage and/or metastatic pancreatic cancer; (2) baseline Karnofsky performance status score ≥50 % (or ECOG performance status <2) and adequate renal, hematological, hepatic, and cardiac functions; (3) aged over 18 years; and (4) without antitumor therapy within 6 months before study. And the exclusion criteria were studies without a full text and non-published conference abstracts. For duplicated literature reports, the most comprehensive ones were selected. Also, other reports might be supplemented.

### Statistical analysis

All analyses were performed strictly with Stata version 12.0 software (Stata Corporation, College Station, Texas, USA). The primary end points included OS. Six-month and 1-year survival rates, ORR, CBR, TTP/PFS, and toxicities were used as second end points. Relative risk (RR) was calculated with a method for dichotomous data and weighted mean difference (WMD) for continuous outcomes and pooled across studies using the DerSimonian and Laird random effects model [13]. The hazard ratios (HRs) with 95 % confidence intervals (CIs) were estimated directly or indirectly from the reported data. And the OS and PFS were measured by HR in this study. The *χ*^2^-based *Q*-test and *I*^2^ statistics were used to assess heterogeneity of studies. If there were statistical differences in terms of heterogeneity (*I*^2^ > 50 %, *p* < 0.10), a random effects model was selected; otherwise, a fixed effects model was used. Forest plots for each meta-analysis were used to present the raw data (means, SDs, and sample sizes) for each arm of the study potential. Publication biases were ascertained by visually inspecting funnel plots for ORR analysis. The relative symmetry of individual study estimates was assessed around overall estimate, followed by Begg’s and Egger’s tests.

## Results

### Literature search

**Fig. 1:**
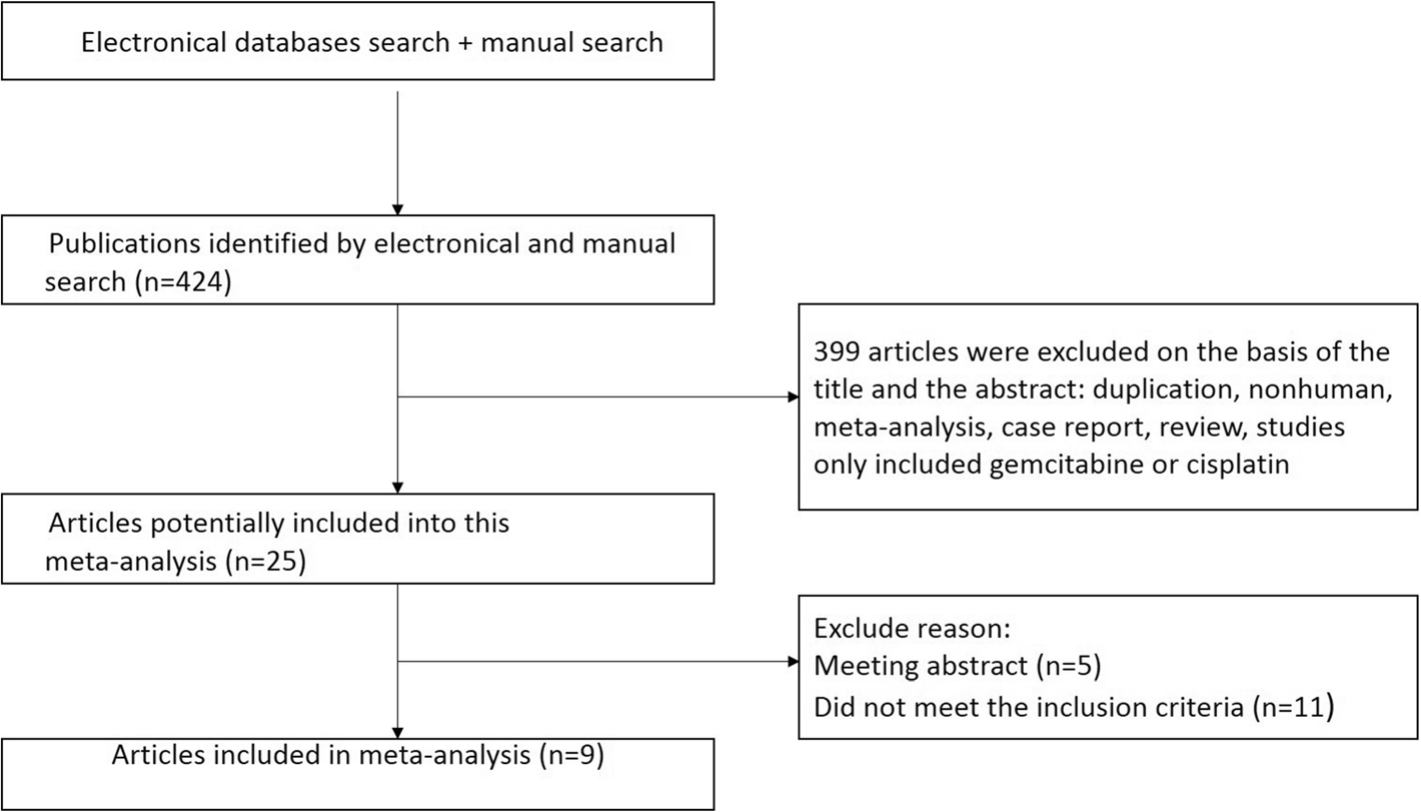
Flowchart of the search strategy

A total of 424 potentially relevant publications were identified by initial electronic searches. After duplicating and reviewing the full texts, only nine articles [2, 14–21] were eligible for this meta-analysis (Fig. 1). A total of eight randomized controlled trials and one retrospective study compared GemCis with Gem alone in advanced and metastatic pancreatic cancers were enrolled in this study.

The basic characteristics of included studies are summarized in Table 1. A total of 1354 patients from nine articles were analyzed. Among them, 725 patients were allocated to the GemCis group and 629 patients in the Gem-alone group. Their median ages were similar at around 45 to 69 years old. And the gender ratios of GemCis and Gem alone were 473/253 and 391/258, respectively. No gender differences existed between trials according to patient characteristics. The values of ORR were extracted from each of the eight trials and 1-year survival rates from seven trials. Only a few trials provided the values of OS, 6-month survival, and CBR.

**Table 1:** Characteristics of nine included trials in the meta-analysis

Studies	Year	Publication type	Inclusion period	Total number	Patients		Median age (range)		Male/female		Jade score
					GemCis	Gem	GemCis	Gem	GemCis	Gem	Gem
Colucci	2002	Phase 3	NA	107	53	54	60 (33–71)	63 (43–75)	35:18	27:27	3
Wang	2002	RCT	July 2000 to May 2001	42	22	20	65 (37–76)	57 (35–60)	15:7	14:6	NA
Heinemann	2006	Phase 3	December 1997 to January 2002	190	95	95	66 (37–82)	66 (43–85)	63:33	59:36	4
H. Palmer	2007	Phase 2	November 1999 to May 2003	50	26	24	66 (47–78)	66 (40–79)	13:13	13:11	3
Sun	2007	RCT	January 2003 to February 2006	53	27	26	55	58	17:10	15:11	3
Colucci	2010	Phase 3	April 2004 to April 2007	400	201	199	63 (37–75)	63 (37–75)	125:76	133:86	4
Inal	2012	Retrospective patient series	September 2006 to March 2011	406	250	156	57	63	175:75	98:58	0
Liu	2012	RCT	May 2005 to July 2008	60	30	30	48 (23–74)	45 (20–69)	13:17	14:16	3
Chao	2013	RCT	February 2000 to December 2002	46	21	25	69 (47–81)	69 (46–83)	17:4	18:7	3

*NA* not available

### Overall survival

**Fig. 2:**
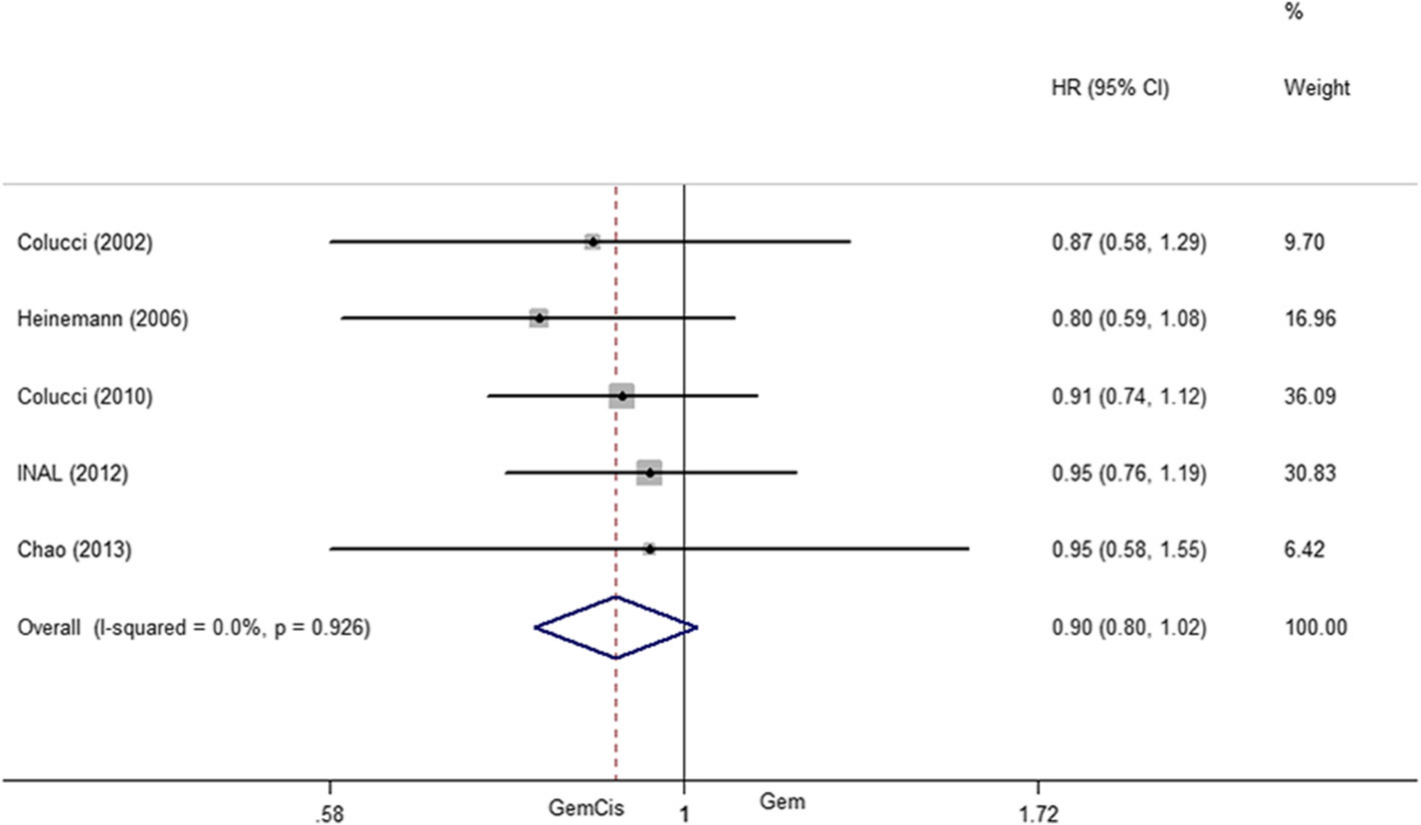
Overall survival between patients treated with GemCis versus Gem

Eight studies reported the median overall survival, ranging from 4.8 to 22 months. The HRs of OS were calculated or acquired from five studies. After pooling the data, no heterogeneity among the studies (*I*^2^ = 0, *P* = 0.926) was found; therefore, a fixed model was employed for meta-analysis of HR. The overall meta-analysis revealed that HR was lower for the patients treated with GemCis than with Gem alone. However, no difference was found between the two groups (HR = 0.90, 95 % CI 0.80–1.42, *P* = 0.10) (Fig. 2).

### Six-month and 1-year survival rates

**Fig. 3:**
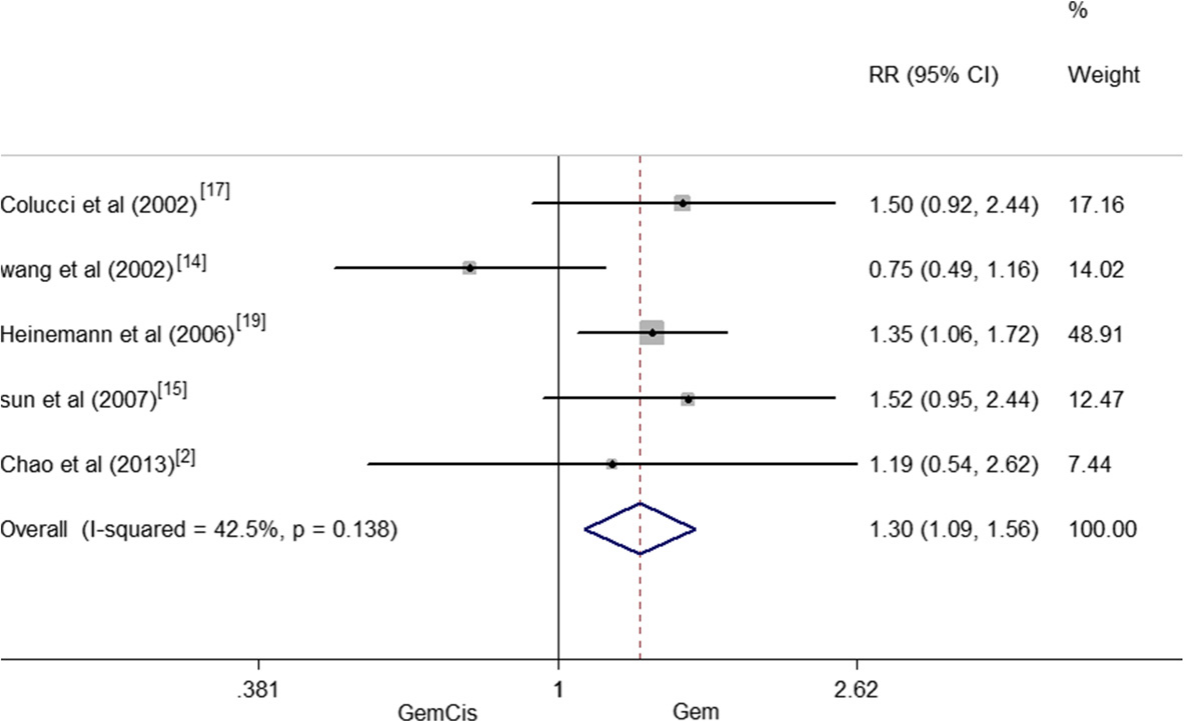
Six-month survival rate between patients treated with GemCis versus Gem

The relevant 6-month survival data were reported for five trials. And a total of 428 patients from these five trials, 213 from the GemCis group and 215 in the Gem-alone group, were included into this meta-analysis. The results showed a general trend of higher 6-month survival rate in the GemCis group than in the Gem-alone group (RR = 1.303, 95 % CI 1.09–1.56, *P* = 0.004). And a small significant heterogeneity existed (*I*^2^ = 42.5 %, *P* = 0.138) (Fig. 3).

**Fig. 4:**
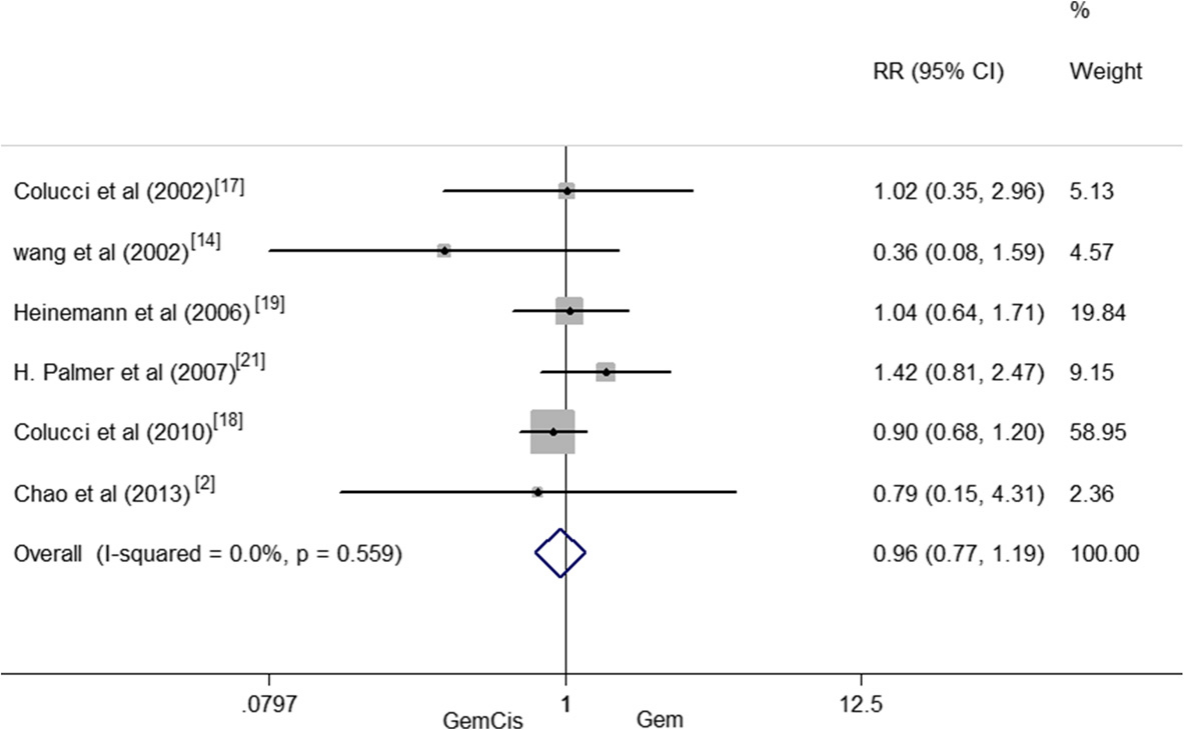
One-year survival rate between patients treated with GemCis versus Gem

Eight hundred twenty-six patients from six randomized controlled trials reported the 1-year survival data. There was no statistically significant inter-group difference that existed in the 1-year survival rate (RR = 0.96, 95 % CI 0.77–1.19, *P* = 0.68). However, no significant inter-group heterogeneity existed in the 1-year survival rate (*I*^2^ = 0.0 %, *P* = 0.559) (Fig. 4).

### Overall response rate

**Fig. 5:**
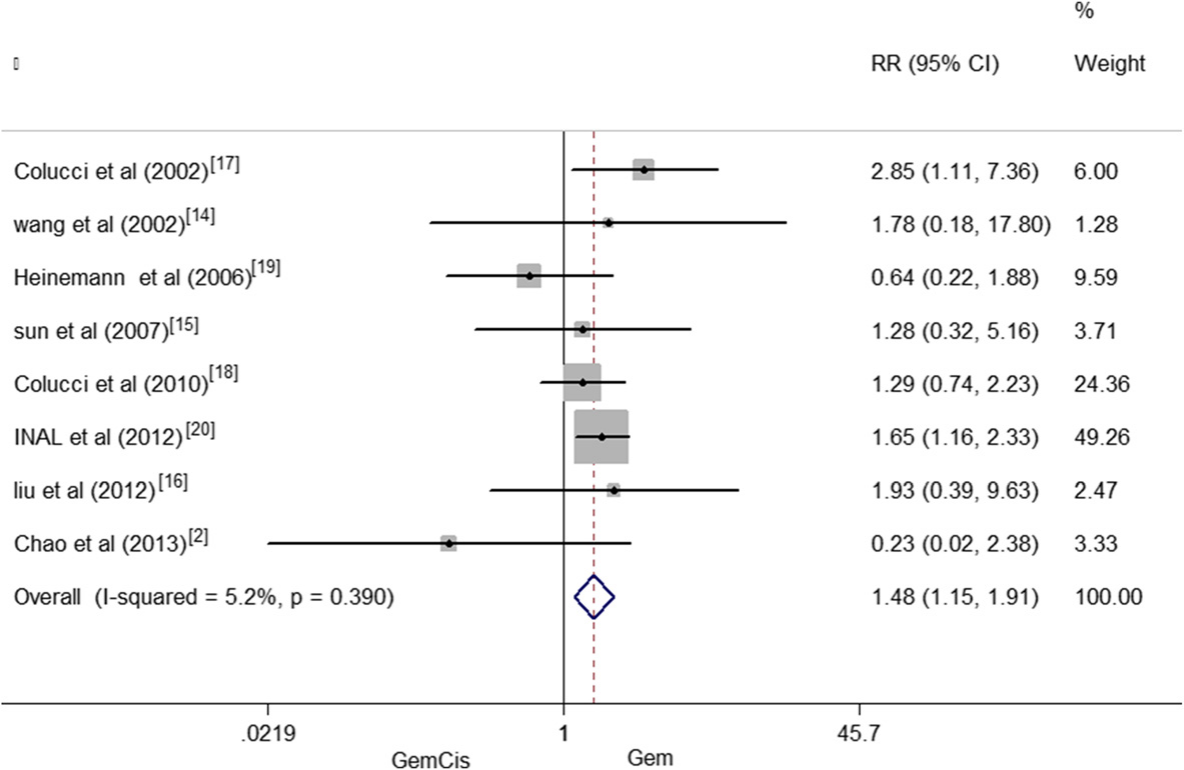
Overall response rate between patients treated with GemCis versus Gem

Eight eligible studies reported the values of ORR. The result of the test for heterogeneity of the therapeutic effect was not significant (*I*^2^ = 5.2 %, *P* = 0.390). So, a fixed effects model was employed. Significant differences existed between GemCis and Gem-alone groups. And the meta-analysis revealed that the combination group was linked with higher ORR than the Gem-alone group (RR = 1.48, 95 % CI 1.15–1.91, *P* = 0.003) (Fig. 5).

### Clinical benefit rate

**Fig. 6:**
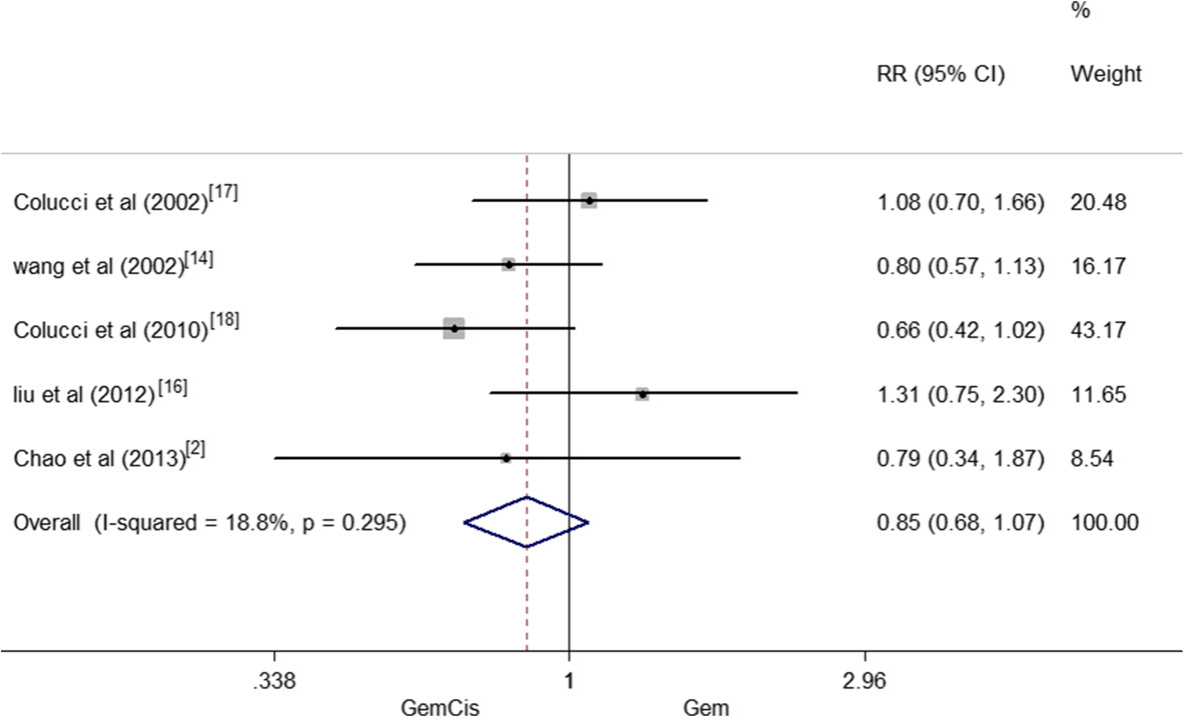
Clinical benefit rate between patients treated with GemCis versus Gem

CBR was reported for five studies involving a total of 578 patients. There were no significant differences that existed between the GemCis and Gem-alone group (RR = 0.85, 95 % CI 0.68–1.07, *P* = 0.175). And small heterogeneity was seen between the two groups regarding the outcome of CBR (*I*^2^ = 18.8 %, *P* = 0.295) (Fig. 6).

### Time to progression/progression-free survival

PFS was defined as the time from random assignment until death or an evidence of tumor progression [19]. And TTP denoted the time from the date of an initial dose of study drug to the observation date of initial disease. In most cases, PFS was rather close to TTP; therefore, the values of TTP and PFS could be co-analyzed [22].

**Fig. 7:**
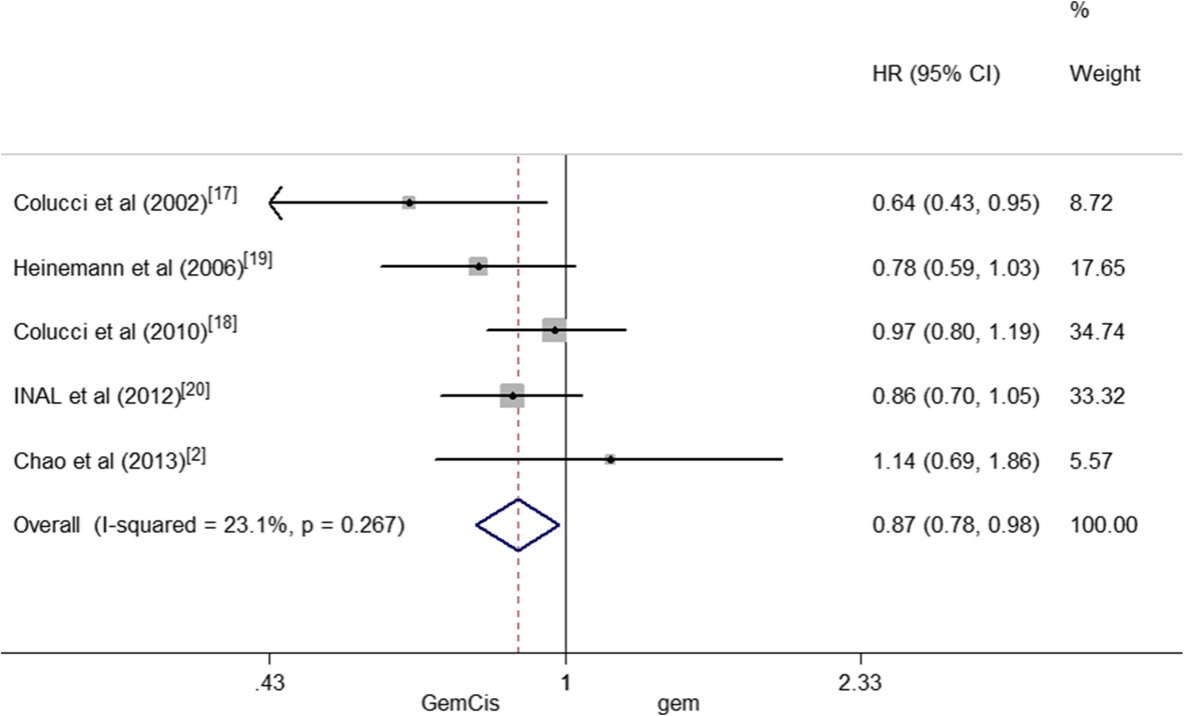
TTP/PFS between patients treated with GemCis versus Gem

Five trials reported the values of TTP/PFS. And 749 patients from the five studies were divided into two groups of GemCis (*n* = 419) and Gem alone (*n* = 330). Overall, there was a significant increase in the TTP/PFS when the GemCis is compared with the Gem group (HR = 0.87, 95 % CI 0.78–0.98, *P* = 0.022). And the heterogeneity between the two groups regarding the outcome of TTP/PFS was low (*I*^2^ = 23.1 %, *P* = 0.267) (Fig. 7).

### Toxicity

The grade III/IV toxic effects of chemoradiotherapy were summarized in the table. As shown in Table 2, neutropenia (18.4 %) was the most common toxicity of the two arms. And the incidence of the other six toxicities was under 10.0 %. After data pooling, there was no evidence of heterogeneity except for thrombocytopenia and diarrhea while the *I*^2^ of diarrhea was 43 % and that of thrombocytopenia was 70.7 %, respectively (Table 2). So, six toxic events used fixed model except thrombocytopenia. As shown in Table 2, as compared with Gem alone, GemCis significantly increased the incidence of neutropenia, anemia, nausea, and vomiting, while showing no difference in the incidence of leukopenia, thrombocytopenia, and diarrhea between the two groups, but GemCis shows a high incidence compared with Gem (Table 2). After data pooling of the seven toxicities, we found that GemCis acquired higher toxicity that Gem alone (RR = 2.164, 95 % CI 1.837–2.549, *P* = 0.000).

**Table 2:** Toxicity of GemCis and Gem

Toxicity	GemCis *n*/*N*	Gem *n*/*N*	RR	95 % CI	*I* ^2^	*P*
Leukopenia	29/603	20/518	1.496	0.865–2.586	0	0.801
Neutropenia	124/529	52/442	2.02	1.493–2.732	0	0.734
Thrombocytopenia	68/552	28/465	1.871	0.724–4.831	70.70 %	0.017
Anemia	72/624	29/537	2.022	1.336–3.060	0	0.591
Nausea	80/624	25/537	2.492	1.629–3.811	0	0.892
Vomiting	59/552	15/465	3.051	1.773–5.253	0	0.773
Diarrhea	32/603	13/518	1.82	0.961–3.446	43 %	0.153

*RR* relative ratios, *CI* confidence interval

### Publication bias

**Fig. 8:**
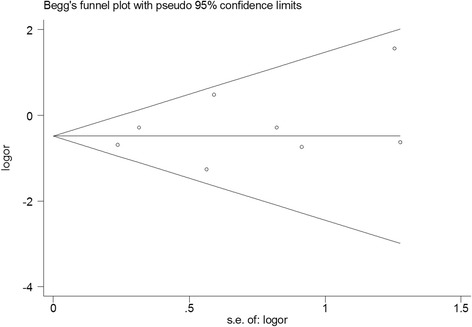
Begg’s funnel plot of publication bias test

Begg’s funnel plot and Egger’s test were performed to assess the publication bias of the selected studies for ORR analysis. The shape of the funnel plots did not show obvious evidence of asymmetry (*P* = 0.71 for ORR, see Fig. 8). The Egger’s test did not show any significant publication bias (*P* = 0.39 for ORR).

## Discussion

Pancreatic cancer is one of the deadliest cancers, with an overall 5-year survival rate of no more than 5 % [23, 24]. Owing to several factors, rapid proliferation of pancreatic cancer cells, aggressive local invasion, metastasis, high local recurrence rate, and resistance to most forms of treatment, the prognosis of pancreatic cancer is still poor [25]. The early stage of pancreatic cancer is usually clinically silent, and we can detect the disease only after it invaded the surrounding tissues or metastasizes to a distant organ [4]. So, most of the patients with pancreatic cancer often lost the opportunity to be resected when diagnosed. Therefore, palliative chemotherapy becomes an option for those patients. However, over the last decades, the treatment of gemcitabine had achieved some great success. In 1997, it was reported that single-agent gemcitabine yielded higher rates of CBR and survival [7]. Afterward gemcitabine has become a mainstay treatment for metastatic pancreatic cancer [25]. Recently, the Gem-based combination therapy was more recommended, due to the limited survival benefit of Gem monopoly. According to the published meta-analysis, Gem-based combination therapy significantly improved OS. However, the advantage was limited due to a higher toxicity. It was suggested that the prescription of Gem-based combination regimens should be selected [26]. Another meta-analysis revealed that polychemotherapy significantly improved OS, PFS, and response rate compared with Gem alone [6]. Cisplatin is a well-known anti-cancer drug, and it is one of the most effective and widely used chemotherapeutic drugs for the treatment of several types of solid tumors, particularly testicular or ovarian cancer patients [27]. Whether GemCis can achieve a better benefit than Gem alone is debated.

In the present meta-analysis, we examined the therapeutic efficacy and safety of Gem-plus-cisplatin-based versus Gem alone for pancreatic cancer. Several clinical studies suggested that a combined use of GemCis was feasible, and it could improve ORR, PFS, and OS [8, 19–22]. According to the included articles in our meta-analysis, the median OS of patients treated by GemCis ranged from 5.5 to 22 months. After pooling the data, it appeared that GemCis may lower the HR for OS than Gem alone; however, no difference was found and and the heterogeneity was existed (*I*^2^ = 0, *P* = 0.926); the heterogeneity that was not found may result from the same chemotherapy medicine. The TTP/PFS reported by the existing studies varied from 3.6 to 10.4 months in GemCis regimen and from 2.0 to 12.2 months in Gem. The pooled HR for TTP/PFS performed by our analysis was 0.87, indicating a 13 % reduction in the risk of death in patients treated with GemCis regimen. The results were also consistent with those of Heinemann’s study. Comparing GemCis with Gem alone, OS and PFS/TTP of GemCis were both superior to those of Gem alone [19]. Yet, absolute improvements of OS and PFS/TTP were marginal. For the 6-month survival rate, GemCis achieved a statistically significant improvement for Gem alone and GemCis had a higher 6-month survival rate of 30 % than Gem monopoly. It was suggested that the combination group could prolong short-term survival rate. Nevertheless, no significant inter-group difference existed in the 1-year survival rate. The short-term 6-month survival rate was consistent with that of Banu et al. [28]. However, the difference in the 1-year survival of two studies might be due to the fact that the combination group of the Banu study contained different drugs. Based upon the above results, GemCis might achieve better outcomes in short-term survival. However, the long-term survival remained inconclusive. Thus, Gem plus cisplatin is recommended over Gem alone.

In the present study, GemCis significantly increase ORR by 48.2 % as compared with Gem alone. Two recent meta-analyses of Gem-plus-fluorouracil versus Gem alone indicated that the combination group significantly improved the outcome of ORR [29, 30]. And it was suggested that cisplatin or fluorouracil-plus-Gem might achieve synergistic effects. Another meta-analysis also revealed that Gem-based combination increased 51 % of ORR than single-agent Gem; moreover, combinations of Gem-plus-platinum salts improved ORR by 77 % as compared with Gem alone [31]. CBR was reported in five of nine studies, and it was lower in the combination group than in Gem-alone group reported in two studies [16, 17]. However, three other articles were on the contrary [14, 15, 18]. Our meta-analysis revealed no significant difference in CBR between GemCis and Gem. One of the most important reasons is that CBR was appraised by pain, functional impairment, and weight loss [17, 18]. However, toxicity and subjective feeling might also play some contributory roles.

In our meta-analysis, we found that GemCis was associated with high incidence of adverse events. Pooled toxicity data significantly increased by 116 % in GemCis versus Gem alone (RR = 2.164, 95 % CI 1.837–2.549, *P* = 0.000). This result was in accordance with another meta-analysis comparing Gem-plus-fluorouracil versus Gem alone [29]. Although the present meta-analysis demonstrated a statistically significantly greater incidence of grades III/IV neutropenia, anemia, nausea, and vomiting in the GemCis group, the PFS, 6-month survival, and ORR, however, significantly increased may make the toxicity generally tolerable and reversible. In our study, some toxicities were excluded for meta-analysis (e.g., mucositis, platelets, and fever) due to the different schemes for safety assessment. Thus, more studies are required for further clarifications.

Some limitations of the present meta-analysis should be acknowledged. Firstly, this meta-analysis only drew upon published data rather than individual patient profiles. Secondly, the sample size was too small to have a sufficient statistical power for the efficiency and safety of pancreatic cancer between GemCis and Gem. Therefore, more studies with larger sample sizes are needed. Third, different doses of cisplatin yielded divergent outcomes.

## Conclusions

The present study meta-analysis revealed a significant improvement in the 6-month survival rate, PFS/TTP, and ORR of pancreatic cancer. However, no significant difference existed in OS, 1-year survival, and CBR. The incidence of grade III/IV toxicity was higher for GemCis than for Gem alone. Yet, the incidence of adverse events for GemCis remained generally tolerable. In conclusion, a combined use of Gem and cisplatin is superior to Gem alone as an alternative chemotherapy for pancreatic cancer. However, owing to the above limitations, more convincing studies are warranted.

## Abbreviations

CBR: clinical benefit rate
CI: confidence interval
Gem: gemcitabine
GemCis: gemcitabine plus cisplatin
HR: hazard ratio
ORR: overall response rate
OS: overall survival
RR: relative risk
TTP/PFS: time to progression/progression-free survival
